# Strategies to Dissect Host-Microbial Immune Interactions That Determine Mucosal Homeostasis vs. Intestinal Inflammation in Gnotobiotic Mice

**DOI:** 10.3389/fimmu.2020.00214

**Published:** 2020-02-18

**Authors:** Allison R. Rogala, Akihiko Oka, R. Balfour Sartor

**Affiliations:** ^1^Division of Comparative Medicine, Department of Pathology, University of North Carolina at Chapel Hill, Chapel Hill, NC, United States; ^2^Center for Gastrointestinal Biology and Disease, University of North Carolina at Chapel Hill, Chapel Hill, NC, United States; ^3^Departments of Medicine, Microbiology and Immunology, University of North Carolina at Chapel Hill, Chapel Hill, NC, United States

**Keywords:** gnotobiotic and conventional mice, mucosal homeostasis, microbiota, fecal microbial transplant (FMT), germ-free (GF), intestinal inflammation, inflammatory bowel disease (IBD)

## Abstract

When identifying the key immunologic-microbial interactions leading to either mucosal homeostasis in normal hosts or intestinal inflammatory responses in genetically susceptible individuals, it is important to not only identify microbial community correlations but to also define the functional pathways involved. Gnotobiotic rodents are a very effective tool for this purpose as they provide a highly controlled environment in which to identify the function of complex intestinal microbiota, their individual components, and metabolic products. Herein we review specific strategies using gnotobiotic mice to functionally evaluate the role of various intestinal microbiota in host responses. These studies include basic comparisons between host responses in germ-free (GF), specific-pathogen-free or conventionally raised wild-type mice or those with underlying genetic susceptibilities to intestinal inflammation. We also discuss what can be learned from studies in which GF mice are colonized with single wild-type or genetically-modified microbial isolates to examine the functions of individual bacteria and their targeted bacterial genes, or colonized by multiple defined isolates to determine interactions between members of defined consortia. Additionally, we discuss studies to identify functions of complex microbial communities from healthy or diseased human or murine hosts via fecal transplant into GF mice. Finally, we conclude by suggesting ways to improve studies of immune-microbial interactions using gnotobiotic mice.

## Introduction

Gnotobiotic rodents are extremely helpful to dissect functional complexity of host/microbial interactions in mucosal immunology and immune-mediated inflammation. This complexity exists at several levels: (1) the incredibly diverse intestinal microbiota that are composed of myriad bacteria, viruses, fungi, and archaea with longitudinal anatomical diversity from the mouth to the distal colon; (2) the interacting innate and adaptive immune responses in the various intestinal compartments that include bone marrow-derived cells, epithelial and mesenchymal cells; lymphoid cells that are “educated” centrally in the thymus (T cells), bone marrow (B cells) spleen (both T and B cells) as well as within the intestine in gut-associated lymphoid aggregates, mesenteric lymph nodes (MLN), lamina propria (LP), and intraepithelial niches; and (3) individualized genetically-determined variable immunologic responses in different hosts. Gnotobiotic animal models can reduce this complexity by isolating functions of specific microbial strains, specific genes within these strains, microbial metabolites, simple groups of related or unrelated microbial strains, and defined consortia, as well as functional consequences of fecal transplants from experimental animal or human donors with various clinical phenotypes or diseases. These microbial manipulations in gnotobiotic recipients provide elegant tools to address several important unresolved questions in disease pathogenesis and clinical outcomes (Table 1). Gnotobiotic animal models provide opportunities to answer these clinically important questions by allowing investigators to precisely manipulate both the microbial environment and host genetic and immunologic pathways to dissect functions of individual microbial strains, their functional genes and metabolites, broad microbial communities as well as host immunologic pathways and genetic predisposition to disease.

**Table 1:** Clinically relevant questions that can be answered by gnotobiotic studies.

1.	Is dysbiosis the cause or consequence of intestinal immune-mediated inflammation/obesity/metabolic syndrome?
2.	What accounts for individual outcomes in disease course and response to therapies?
3.	Can manipulation of microbial composition or function alter disease activity or outcomes?

This review emphasizes the microbial side of this equation, yet provides targeted examples of different genetically-determined host responses and key regulatory and effector immune pathways that mediate the biologic function of illustrated microbial strains. Rather than trying to comprehensively catalog microbial induction of homeostatic and effector immune functions that prevent or mediate intestinal inflammation that are addressed in other comprehensive reviews (1–3), we emphasize strategies using gnotobiotic rodents to assess various functions of microbial species, their functional pathways and metabolites, and defined consortia and complex microbial communities derived from healthy or diseased subjects (Table 2). Gnotobiotic studies necessarily evaluate a defined microbial strain or combination of strains, so results with a specific strain may not represent the diversity of functions of the relevant species. Therefore, readers must carefully interpret results of a study that uses a single or group of bacterial strains and investigators should choose clinically relevant strains and fully define their characteristics in publications.

**Table 2:** Strategies to functionally analyze microbial constituents, genes, and metabolites in gnotobiotic mice.

**Microbial target**	**Strategy**
Complex resident microbiota	GF vs. SPF, SPF, or human FMT → GF
Active components of complex microbial groups	Progressive deconvolution, functional screening
Defined consortia (intra- and trans-kingdom)	Colonize GF—groups of deconvoluted strains
Single strains	Monoassociation compared with GF or SPF
Functional genes	Monoassociate isogenic strains: wild-type and deletion, transgenic, complemented mutants
Metabolic pathways	Substrate availability, isogenic gene deletion, provide agonist to GF mice, antagonists, host receptor mutants.

*GF, germ-free; SPF, specific pathogen-free; FMT, fecal microbial transplant*.

**Figure 1:**
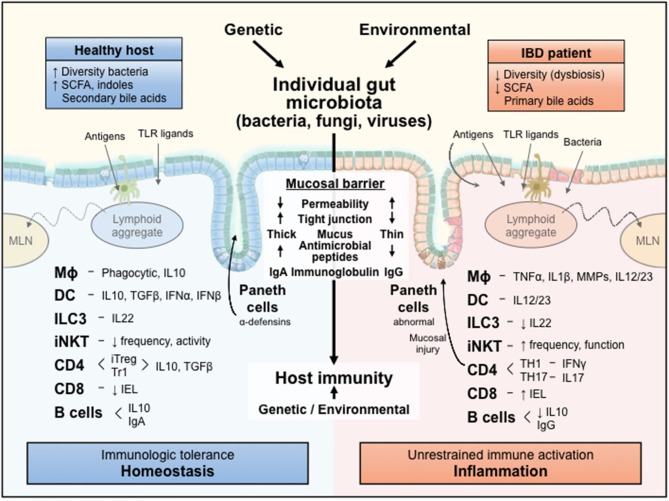
A conceptual framework for the influences of an individual's resident microbiota on mucosal immune responses. The diverse microbiota and their metabolic products are selectively sampled by the intact mucosa of a healthy host to activate regulatory immune responses that mediate homeostasis. In contrast, the dysbiotic microbiota and their immunologically active components leak through the permeable mucosa of a genetically susceptible host to stimulate unrestrained aggressive effector immune responses that cause inflammation and tissue injury. IL, interleukin; TLR, Toll-like receptor; MLN, mesenteric lymph node; Mϕ, macrophage; DC, dendritic cell; ILC3, innate lymphoid cell 3; iNKT, invariant natural killer T cell; CD, cluster of differentiation; IEL, intraepithelial lymphocyte; SCFA, short chain fatty acids; Ig, immunoglobulin; IFN, interferon; TNF, tumor necrosis factor; MMP, matrix metalloprotease; iTreg, inducible T regulatory; TGF, transforming growth factor; T_H_, T helper; T_R_1, T regulatory 1.

Figure 1 and numerous detailed reviews (1–10) provide a conceptual framework for our discussion of dissecting microbial-immune reactions by gnotobiotic investigations. Each individual host, whether rodent or human, has a unique resident intestinal microbiota that is determined in part by the host's genetic background, but to a larger extent by environmental factors (diet, housing conditions, exposure to infections and antibiotics, etc.) (11). The highly diverse microbiota of healthy hosts activate predominately regulatory immune pathways mediated by multiple interacting types of innate and adaptive immune cells and help maintain a healthy mucosal barrier determined by a relatively impenetrable mucus layer that concentrates secreted neutralizing immunoglobulins and a variety of antimicrobial peptides and lectins, as well as epithelial-tight junctions. This healthy mucosal barrier and regulatory immune function mediate mucosal homeostasis and immunologic tolerance to resident microbiota, yet permit rapid activation of innate and adaptive immunity to clear invasive microbial pathogens that breach the mucosal barrier. In contrast, the dysbiotic microbiota and increased uptake of microbial components across the permeable mucosa of hosts susceptible to chronic inflammation activate dysregulated effector immune responses that promote tissue injury and further perpetuate uptake of immunologically active bacterial toll-like receptor (TLR) agonists, antigens and metabolites that drive unrestrained chronic immune-mediated inflammation, such as Crohn's disease (CD), ulcerative colitis (UC) and chronic experimental colitis (3, 4).

Genetic susceptibility is a key element in an individual host's response to resident microbiota, as illustrated by differential outcomes when identical complex microbiota, single bacterial strains or defined bacterial consortia are transferred to normal vs. genetically engineered germ-free (GF) recipients (12–15). Similar different outcomes were observed when dysbiotic or high diversity fecal specimens are used to colonize GF wild-type (WT) or colitis models indicating the primary role of eubiosis or dysbiosis in driving homeostasis or inflammation. Many of the genes that are manipulated to confer risk to inflammation function as immune regulators, showing the importance of immune regulation in determining an individual host's response to resident microbiota (15–17). Thus, microbe/immune interactions are complex responses with important influences from both host genetically programmed immune pathways and microbial composition and function. We discuss strategies to use the power of gnotobiotic rodents to dissect the functional role of microbial components, using highly selected examples from published studies.

### Homeostatic/Protective Immune Responses

The complex microbiota in normal hosts help stimulate protective homeostatic immune responses that inhibit effector inflammatory response to resident microbial antigens and innate immune agonists (3, 9, 10). We discuss gnotobiotic techniques that use the strategies outlined in Table 2 to dissect the function of complex microbiota and their components.

#### Evaluating Immunoregulatory Functions of Complex Resident Microbiota in GF vs. SPF and Fecally Transplanted Mice

GF mice exhibit less developed innate and adaptive immune functions, with smaller Peyer's patches, IgA levels, LP lymphoid populations and M_1_/M_2_ tissue macrophages relative to conventionally raised mice (18–22). We demonstrated a >8-fold increase in the number of colonic LP FoxP3^+^ CD4^+^ cells in C57BL/6 mice born specific pathogen-free (SPF) compared with those maintained GF (23). FoxP3^+^ RORγT^+^ inducible regulatory T cells (Tregs) accounted for the majority of the increased CD4^+^ Tregs in SPF mice. Colonizing GF mice with SPF mouse microbiota increased the frequency of FoxP3^+^ and FoxP3^+^ RORγT^+^ LP CD4^+^ cells to levels very close to those of mice raised under SPF conditions, with inducible Tregs detected within 3 days. In parallel, the LP of GF IL-10^egfp^ reporter mice contained <1/3 the number of IL-10-producing CD45^+^ cells relative to SPF mice, confirmed by colonic tissue levels of IL-10 protein and mRNA expression. Transplant of SPF feces → GF mice increased numbers of colonic LP IL-10-producing cells, primarily CD25^neg^ CD4^+^ T cells and CD19^+^ B cells, and colonic IL-10 concentrations after 7 days almost to levels seen in SPF-housed mice. Using cells from SPF TLRs, MyD88 and phosphatidylinositol 3-kinase (PI3K) knockout (KO) mice, TLR agonists and pharmacologic antagonists, we demonstrated that induction of IL-10 in GF B cells involves TLR2 signaling through MyD88 and the PI3K subunit p110δ (23). Faith et al. similarly reported that GF mice have decreased levels of FoxP3 and inducible RORγT^+^ Tregs relative to SPF mice and that transplant of feces from healthy human controls to GF mice for at least 28 days substantially increased the number of Tregs and inducible FoxP3^+^ RORγT^+^ Tregs in both the ileum and colonic LP (16). These experiments demonstrate the rapid induction of regulatory T and B cells in the distal intestine following exposure to complex resident microbiota in normal hosts. Multiple other studies show the importance of resident microbiota in activating homeostatic innate lymphoid cells (ILC) (24), intraepithelial lymphocytes (25) and the microbicidal C-type lectin, RegIIIγ, a product of epithelial cells, particularly Paneth cells (26). RegIIIγ activation is dependent on MyD88 and mediates spatial segregation of gut microbiota from the intestinal epithelium (27). For example, GF C57BL/6 mice have decreased numbers of small intestinal LP ILC subset 3 (ILC3) cells and fecal transplants or selective colonization of lymphoid tissue-associated bacteria induced IL-10 production by small intestinal dendritic cells (DC) and IL-22 by ILC3 (28). In addition, Gury-Ben Ari et al. used GF and broad spectrum antibiotic-treated mice to show that resident microbiota drive differential transcriptional and epigenetic responses in ILC subsets 1, 2, and 3 and cluster-specific ILC transcriptional responses by single cell analysis (24). These cell-specific effects are supported by the complementarity and redundancy of subsets of IL-22-producing colonic ILC3 cells (29).

#### Establishing Functional Consequences of Resident Microbial Alterations Using Fecal Microbial Transplant (FMT)

Transplanting native complex resident microbiota can examine functional consequences of dysbiosis associated with clinical phenotypes. These functional studies that directly address the “cause vs. consequence” controversy of dysbiosis are typically performed in disease-susceptible GF recipients (see Inflammatory Immune Response section below). However, Britton et al. recently compared protective to inflammatory intestinal T cell profiles in GF C57BL/6J mice that received FMT from healthy donors or patients with either CD or UC (16). Although the frequency of total FoxP3^+^ cells in the ileal and colonic LP were not different with the two donor sources, the number of inducible RORγT^+^ Tregs was higher following colonization with healthy control microbiota compared with either UC or CD patient FMT. Reciprocally, the frequency of effector TH_17_ and TH_1_ cells was higher with the inflammatory bowel diseases (IBD) microbiota. Because 16S rRNA sequencing analysis did not show substantial differences in the healthy control and IBD fecal specimens, the functional differences evident after FMT from the various donor sources are likely driven by species-level alterations and functional differences (for example metabolite production) between the donor sources.

Chung et al. colonized GF Swiss Webster mice with mouse, normal human and rat fecal microbiota to explore recipient responses to microbial colonization from different donor species (30). In the offspring of these mice the murine microbiota induced larger numbers of small intestinal LP intraepithelial CD4^+^ and CD8^+^ T cells, as well as LP FoxP3^+^ CD4^+^ T cells compared with healthy human microbiota-colonized mice, which exhibited T cell profiles similar to GF mice. The deficient number of small intestinal T cells was partially restored by segmented filamentous bacteria (SFB) monoassociation of GF mice. These LP T cell alterations were accompanied by parallel differences in DC numbers in Peyer's patches and MLNs following murine or human FMT, providing a potential mechanism of differential T cell activation and proliferation. Differential activation of anti-microbial innate responses as measured by RegIIIγ expression followed the pattern of small intestinal T cells. Because other investigators, including ourselves, have clearly demonstrated functional colonic immune cell activity of transferring healthy human fecal microbiota to GF C57BL/6 mice (16, 31, 32), methodologic and anatomical differences may explain the divergent outcomes. Chung et al. used the offspring of FMT-colonized GF mice, so their study population was exposed to microbiota transferred naturally by maternal-fetal transfer. Early life exposure to complex microbiota, particularly within the first 4 weeks of life, shape murine mucosal immune responses, including invariant natural killer T cells (iNKT) numbers, and subsequent risk of developing experimental colitis and asthma (33, 34). Moreover, the human microbiota in the Chung study were derived from two healthy donors (30), which likely do not represent the highly diverse population of microbes within heterogeneous normal human subjects. These studies were performed in outbred GF Swiss Webster mice, which could have different outcomes from the more commonly used C57BL/6 strain. Finally, these studies were restricted to small intestinal immune cells and associated lymphoid organs, with no data provided on colonic LP cells. Several investigators, including Atarashi and Honda (35) and Faith et al. (36) showed no differences in the percentage of FoxP3^+^ CD4^+^ cells in the small intestinal LP in SPF C57BL/6 mice and ex-GF mice colonized with human microbiota compared with GF controls, yet highly significant increases in colonic LP cells. These results indicate important differences in the ability of transferred human microbiota to activate small intestinal compared to colonic immune cells that must be considered in the design and interpretation of FMT experiments.

One of many examples of transferring a functional phenotype by FMT from KO mice to WT mice is provided by elegant studies by Lamas et al. (37). These investigators noted compositional changes in fecal fungal and bacterial communities in *CARD9*^−/−^ vs. WT mice that were associated with increased susceptibility to dextran sodium sulfate (DSS)-induced colitis. CARD9 is an intracellular adaptor protein that transduces ITAM-associated and MyD88-mediated signals in DC and macrophages. CARD9 deficiency is associated with enhanced susceptibility to candida and other fungal infections in humans. FMT from *CARD9*^−/−^ mice to GF WT recipients increased the recipient's susceptibility to colitis. In addition, transfer of the *CARD9*^−/−^ microbiota to GF recipients decreased expression of protective *IL-22, RegIIIg*, and *RegIIIb* compared to FMT from WT mice. In addition, the *CARD9*^−/−^ fecal transplant was associated with decreased bacterial tryptophan metabolites, such as indole-3 acetic acid that serve as ligands for aryl hydrolase receptors (AhR) that induce IL-22 and protect the mucosal barrier (38–40) (see later Functional Studies section that describes metabolomic mechanisms).

Together these studies demonstrate the opportunity to study functional properties of complex murine and human microbial populations from human or murine hosts with a variety of phenotypes by colonizing GF recipients. This approach is particularly helpful to explore the functional consequences of microbial communities associated with human disease and provides a means to mechanistically dissect the functions of the various microbial subsets that are expanded or contracted in human disease states.

An important issue is the efficiency of engraftment following transplant of human or murine microbial communities to GF recipients. Important variables include pre-reduced or ambient media used to homogenize specimens, oral gavage vs. rectal administration and frequency of colonization. Optimal techniques of gavage transfer of human fecal samples in pre-reduced media to GF mice result in a stable microbiota community after 28 days (31). All bacterial phyla, 11/12 bacterial classes and 58/66 genus level taxa detected in the donor sample were recovered in the recipient mouse feces by 28 days. The gene level taxa missing from the humanized mice were present in very low abundance (0.008% average) in the human donor samples. Diversity in the human donor and murine recipient microbiota was not significantly different (Shannon diversity index 4.53 ± 0.15 (donor) vs. 4.09 ± 0.05 (recipients on mouse chow) and 4.51 ± 0.04 (recipients on Westernized diet). No important differences were seen in freshly prepared vs. frozen human donor fecal aliquots. The Gordon group replicated their initial findings with larger numbers of donors and in different intestinal regions (41). Similarly, our lab documented preservation of human donor microbiota profiles in WT and IL-10^−/−^ mice following two serial gavages 2 days apart using pre-reduced diluent. Feces of IL-10^−/−^ mice recipients of human fecal transplant retained the overall composition of the human donor 2 weeks after fecal transplant (Phyla 100%, Class 100%, and Genus 78%) (32). We acknowledge that all human bacterial species do not colonize the murine gut after fecal transplant; however, transferred human microbiota retain their overall composition and function quite well. Human fecal transplant to GF mice represent a valuable strategy to investigate altered functions of microbiota communities in human disease, a conclusion echoed by Nguyen et al. in their thoughtful review (42). However, improving transfer of low frequency species is an important goal.

#### Dissecting Functional Components of Complex Microbiota and Defined Consortia

Several methods have been developed to identify which subsets of a complex microbiota preferentially induce regulatory immune responses. We highlight two separate strategies that use gnotobiotic mice to identify dominant functional components of active resident microbial populations or defined culture collections of human resident bacterial strains.

##### Identifying protective Clostridium strains in murine and human fecal specimens (reductionist approach)

Atarashi and Honda developed an elegant method to identify a subset of human Clostridium strains that induce colonic Tregs and protect against colitis in multiple models (35, 43). They first demonstrated that resident microbiota activate colonic Tregs by showing that SPF BALB/c, IQI and C57BL/6 mice had substantially higher percentages of LP FoxP3^+^ CD4^+^ cells than did GF mice, but no such differences were observed in the small intestine. Fecal transplant from SPF C57BL/6 mice increased numbers of colonic LP FoxP3^+^ cells in ex-GF recipients. A role for spore-forming Gram positive bacteria, primarily Clostridium species, was established by showing that vancomycin, but not polymyxin B decreased numbers of FoxP3^+^ colonic LP cells and that colonizing GF mice with chloroform-treated SPF mouse feces increased the frequency of colonic FoxP3^+^ cells. A defined consortium of 46 murine Clostridium strains, but not SFB, 3 Lactobacillus strains or *Bacteroides fragilis*, induced Helios^neg^ colonic LP FoxP3^+^ cells in ex-GF BALB/c and IQI mice 3 weeks after colonization. These Clostridium strains primarily consisted of clusters IV and XIVa (*C. leptum* and *C. coccoides* groups, respectively), which are clinically important because they are decreased in human IBD (44). These 46 Clostridium strains stimulated production of TGFβ when cultured *ex vivo* with colonic epithelial cells and induced extrathymic activated (mostly Helios^neg^ and CTLA4^high^) IL-10-producing FoxP3^+^ colonic LP cells in gnotobiotic IL-10^venus^ reporter mice. These 46 Clostridium strains stimulated colonic LP Tregs in SPF mice and attenuated DSS-induced colitis. These studies were then expanded to human Clostridium strains isolated from chloroform-treated feces from a single healthy female volunteer (43). The native and chloroform-treated human samples increased FoxP3^+^ colonic LP cells in colonized GF IQI mice 3–4 weeks after colonization, but the intact human feces also induced IL-17^+^ cells. 2 × 10^4^ diluted stools continued to stimulate colonic Tregs, indicating that the inducing microbial population was abundant and stable since several serial oral inoculations continued to induce colonic Tregs. Inoculation of GF mice with 23 individual strains cultured from the serially passed diluted samples increased colonic Helios^low^ FoxP3^+^ cell percentages similar to the human chloroform-treated samples. The 17 Clostridium strains recovered from the gnotobiotic mice induced colonic Tregs in three gnotobiotic mouse strains, BALB/c, IQI and C57BL/6, as well as selectively colonized ex-GF rats and increased the proportion of colonic LP IL-10^+^, CTLA4^+^, and ICOS^+^ Treg subsets. Subsets of 3–5 Clostridium strains induced colonic Tregs, but less than the entire 17 strain community, suggesting that these strains act additively or synergistically. These 17 strains belong to Clostridium clusters IV, XIVa and XVIII by genomic sequencing. Five of these strains (strains 1, 4, 15, 21, 29 most closely aligned with Clostridium SP14774, *C. hathewayi, C. asparagoforme, Eubacterium fissicatena*, and Lachnospiraceae 3-1-57FAA, respectively) were selectively decreased in UC patients in the MetaHIT database. In SPF mice, the 17 strains stimulated a higher percentage of colonic LP Tregs and attenuated experimental colitis in three separate models (trinitrobenzene sulfonic acid [TNBS], T cell transfer and ovalbumin/adjuvant). Refined subsets of this consortium are now undergoing Phase 1 human studies. These studies show the power of using gnotobiotic mice to discover clinically relevant subsets of human resident microbiota that stimulate protective mucosal immune responses and protect against intestinal inflammation and describe a reductionist approach to identify the dominant functionally active bacterial strains.

##### Combinatorial approach

Faith, Gordon et al. applied a combinatorial approach to identify a broad range of human resident bacterial species that induce colonic Tregs, modulate mouse adiposity and alter cecal metabolites, and created several novel innovations that facilitate similar studies under gnotobiotic conditions outside of standard flexible film isolators (36). These investigators initially screened fecal strains from five different US female volunteers for their relative ability to activate intestinal Tregs by colonizing GF C57BL/6 mice. After 2 weeks, each of the five human microbiota increased frequency of FoxP3^+^ cells in the colonic LP, but not reproducibly in small intestinal LP, MLN and splenic CD4^+^ cells in recipient humanized ex-GF mice compared to GF controls. The investigators anaerobically cultured 17 isolates from one representative donor that represented the four most prominent bacterial phyla in healthy adult humans (Bacteroidetes, Firmicutes, Actinobacteria, and Proteobacteria). Selective colonization of GF mouse recipients with this pool of 17 strains increased the numbers of colonic LP Tregs. They developed a probabilistic tool to guide phenotypic responses to hypothetical combinations of the 17 strains and then tested *in vivo* phenotypes by generating 94 distinct random subsets of 1–17 strains each that were used to gavage a total of 124 GF mice fed a defined polysaccharide diet. This huge undertaking was facilitated by utilizing housing conditions in sterilized filter top cages maintained outside of standard flexible plastic film gnotobiotic isolators for 2 weeks. This design allowed testing of each bacterial strain in at least 46 different subsets. Fifteen of the seventeen strains were reproducibly detected in recipient mice. The total biomass of bacteria was constant in each subset and reached luminal saturation with two or more strains. As few as two different bacterial strains increased levels of colonic LP Tregs equivalent to values achieved by inoculation with the entire uncultivated donor specimen. Monoassociation studies demonstrated highest Treg induction by *Bacteroides intestinalis* and lowest by *Collinsella aerofaciens*, which colonized to low densities. All but two of the 10 monoassociated strains induced higher colonic LP Treg levels than occurred in GF controls, including each of five tested Bacteroides strains and one Parabacteroides strain. In selected mice, the investigators demonstrated greater populations of neuropilin-1 (Nlrp1)^low^ FoxP3^+^ cells in the colonic LP and MLN, but not the spleen, suggesting peripheral activation of Tregs rather than intrathymic expansion.

This innovative study demonstrates that a broad group of resident bacterial strains that extend beyond Clostridium strains, which were not studied, can induce colonic peripherally activated Tregs. The investigators developed out-of-isolator methods to facilitate screening of large numbers of bacterial strains or groups that can be applied to newer individually–ventilated isopositive caging systems in which each cage is equivalent to an isolator.

#### Documenting Protective Activities of Individual Bacterial Strains (Monoassociation and Group Colonization of Gnotobiotic Mice)

Multiple investigators have demonstrated that a variety of individual bacterial species, including *Bacteroides fragilis, Bifidobacterium infantis, Faecalibacterium prausnitzii*, and the previously mentioned consortia of human and murine Clostridium and Bacteroides strains have the capacity to activate protective immune responses, including induction of Tregs (35, 36, 43, 45–47). Although monoassociation strategies are relatively straightforward, we highlight several high impact studies that explore mechanisms of protection to illustrate strategies of how investigators have approached this topic.

##### B. fragilis

Mazmanian, Kasper, Round et al. in a series of elegant studies documented that *B. fragilis* can restore normal mucosal immune responses in GF mice and stimulate Tregs through its capsular polysaccharide A (PSA) (21, 45, 48). *B. fragilis* was chosen for study because it prominently colonizes the distal intestinal tract and produces eight distinct capsular polysaccharides. Several of these polysaccharides are zwitterionic molecules that possess positive and negative charges that stimulate proliferation of CD4^+^ T cells after processing by antigen-presenting cells and presentation on MHC class-II molecules. PSA is the most immunodominant *B. fragilis* polysaccharide. Monoassociation of GF Swiss Webster mice with *B. fragilis* NCTC9343 almost completely restored the deficient number of CD4^+^ cells in the spleens of GF mice to levels found in SPF mice, a function dependent on PSA, since colonization with an isogenic *B. fragilis* PSA-deletion mutant (ΔPSA) failed to correct deficient CD4^+^ cell numbers despite luminal colonization equal to the WT strain (21). WT *B. fragilis* selectively stimulated CD4^+^ T cell proliferation, since colonization had no effect on CD8^+^ T cells and CD19^+^ B cell numbers. Intraperitoneal administration of purified PSA increased numbers of splenic CD4^+^ cells, as did oral gavage of PSA to SPF mice. Orally fed PSA associated with MLN CD11c^+^ DCs. *B. fragilis* and PSA broadly induced CD4^+^ cell responses, including reversing the TH_2_ skewing of GF mice to a TH_1_ profile, as documented by enhanced IFNγ production following monoassociation with WT *B. fragilis*, but not the ΔPSA mutant. Further investigations targeted intestinal FoxP3^+^ Tregs (45). WT *B. fragilis* monoassociation increased intracellular IL-10 production by colonic LP FoxP3^+^ CD4^+^ cells and MLN FoxP3^+^ cells, but the ΔPSA mutant had no effect (45). They explored peripheral differentiation of T cells by adoptively transferring FoxP3^neg^ CD4^+^ cells from FoxP3^egfp^ mice to GF Rag^−/−^ mice. Monoassociating these mice with WT, but not ΔPSA *B. fragilis* induced FoxP3^egfp+^ CD4^+^ cells with intracellular IL-10 staining. Studies in SPF WT and TLR2^−/−^ mice show that purified PSA prevented and reversed TNBS-induced colitis in a TLR2-dependent manner associated with the induction of FoxP3^+^ cells (45).

##### Clostridium species

As described above, Atarashi, Honda et al. extensively studied the function of subsets of murine and human Clostridium species in selectively colonized gnotobiotic mice (35, 43). Colonization of gnotobiotic mice stimulated increased percentages of Helios^neg^ IL-10-producing FoxP3^+^ CD4^+^ cells in the colonic LP, but not in the small intestine.

These selected studies illustrate several approaches that can be used to define the protective functions of selected bacterial strains either alone or in combination. Combined use of genetically modified microbial strains and gnotobiotic KO or transgenic (TG) mice can further isolate the functional interactions of bacterial genes and host pathways that mediate homeostatic immunologic functions in the distal intestine.

#### Functional Studies

Gnotobiotic mouse studies offer an optimal platform to identify microbial-host immune and -dietary interactions that determine mucosal immune homeostasis by selectively manipulating single microbial and host genes and dietary factors. These studies can address the “cause vs. consequence” controversy outlined in Table 1. While host genetic effects can be studied in SPF KO and TG mice, secondary effects of manipulating specific host genes can indirectly affect host microbiota (37). These indirect effects can be mitigated by studying GF or selectively colonized WT, KO or TG mice with single or simplified microbiota. Additionally, metabolic pathways can be examined in the absence of other microbial influences by treating GF mice with purified metabolites or metabolic pathway agonists or alternatively by blocking bacterial metabolite synthesis or host receptors. We provide several examples of studies to illustrate potential strategies for this line of investigation.

##### Genetic manipulation of a bacterial strain

A clear example of genetically manipulating a bacterial strain to examine potential protective immune responses is the role of PSA in mediating immune competence and Treg activation (21, 45). As detailed above, Mazmanian, Kasper, Round et al. monoassociate GF mice with WT or ΔPSA *B. fragilis* to show that deficient numbers of splenic CD4^+^ cells (21) and colonic LP IL-10-producing Tregs (45) in GF mice were increased by WT, but not ΔPSA *B. fragilis*, a response replicated by administering purified PSA to GF mice.

Kasper, Blumberg et al. similarly dissected the role of bacterial sphingolipids in regulating numbers and functions of iNKT cells using targeted deletion mutant *B. fragilis* in monoassociated mice. They initially observed that GF mice exhibited increased numbers of colonic iNKT cells and developed potentiated oxazolone-mediated colitis, which is mediated by iNKT cells (33). Targeted deletion of the geneBF2461, which encodes a bacterial enzyme in the sphingolipid biosynthetic pathway in *B. fragilis* NCTC9343 (ΔSPT) eliminated *in vitro B. fragilis* synthesis of sphingolipids, but not phospholipid production (49). Sphingolipid synthesis was restored to WT levels in the complemented strain (C-delta). The offspring of GF Swiss Webster mice monoassociated with the WT or C-delta *B. fragilis* and SPF mice displayed significantly lower percentages and absolute numbers of colonic iNKT cells than did GF and *B. fragilis* ΔSPT-monoassociated mice. No differences in extracolonic iNKT cells were apparent in the different groups, including thymus, spleen, lung, small intestine, and Peyer's patches, indicating focal activity of *B. fragilis* sphingolipid production in the colon. Importantly, ontogeny studies demonstrated that very early life colonization, within the first 12 days, was necessary to suppress proliferation of colonic iNKT cells, with effects persisting throughout life. The immunoregulatory effects of this suppression of colonic iNKT cell number and function was documented by potentiated oxazolone-induced colitis in ΔSPT-monoassociated mice compared with WT *B. fragilis*-colonized mice. Gavage administration of α-galactosylceramide purified from *B. fragilis* to ΔSPT-monoassociated mice 2–7 days of age replicated endogenous bacterial activity by decreasing colonic iNKT cell proliferation in neonatal mice, decreasing adult colonic iNKT cell numbers and protecting against oxazolone-induced colitis in adult mice compared with vehicle-treated controls. These elegant studies showing homeostatic immunoregulatory effects of sphingolipid produced by *B. fragilis* were validated and their clinical relevance extended by Ramink Xavier's group, who showed that a variety of resident Bacteroides strains, including *B. thetaiotaomicron* and *B. vulgatus*, produced at least 35 unique sphingolipids (50). Importantly, immunoprotective effects of the *B. thetaiotaomicron* sphingolipids were documented by small intestinal and colonic inflammation in gnotobiotic C57BL/6 mice monoassociated with the ΔSPT mutant, with no inflammation observed in WT *B. thetaiotaomicron*-monoassociated mice. This rapid onset of inflammation 3 days after colonization was manifested by colonic goblet cell hypoplasia and increased concentrations of IL-6 and MCP-1 protein levels and tissue macrophages in the colons of ΔSPT-monoassociated mice compared with WT-colonized mice. Of clinical significance, fecal metabolomic studies demonstrated decreased levels of Bacteroides sphingolipids in IBD vs. healthy controls that negatively correlated with fecal calprotectin, a biomarker of inflammation. These findings were striking because host-derived sphingolipids were among the most elevated metabolites in IBD patients (50). Together, these studies demonstrate an important role for Bacteroides sphingolipids in maintaining mucosal homeostasis, adding these metabolic products to a growing list of immunoregulatory metabolites produced by resident bacteria.

#### Metabolic Studies

Bacterial metabolites mediate many of the protective functions of resident bacteria and are notably abnormal in the dysbiosis associated with IBD (51, 52). Metabolites with known immunoprotective activities include the short chain fatty acids (SCFAs), butyrate and propionate, tryptophan metabolites, particularly indoles that serve as AhR ligands, sphingolipids and bile acid derivatives (49, 50, 53–58). However, this is a rapidly developing field with new bacterial metabolites associated with IBD and yet unknown functions being frequently described (51, 52, 59). We provide several examples of how gnotobiotic mice can help dissect functions of two key immunoprotective bacterial metabolites, SCFA and indoles, since we discussed functional studies of sphingolipids in a previous section.

##### SCFAs

SCFAs are reproducibly decreased in feces of IBD patients (51) and have key homeostatic functions, including providing the primary metabolic fuel for distal colonic epithelial cells and inducing Tregs. Two pivotal studies used gnotobiotic mice to help identify the role of intestinal butyrate and propionate in the induction of peripherally-activated Tregs (53, 54). The Honda group previously showed that inducible Tregs were deficient in the colons of GF mice, but were expanded by selective colonization with chloroform-resistant spore-forming Clostridium species (35). Furusawa et al. subsequently demonstrated that high fiber diets enhanced inducible colonic Tregs in ex-GF IQI mice colonized with murine chloroform-treated feces to a greater extent than did low fiber diets (53). Both diets increased numbers of colonic inducible Tregs when administered to Clostridium-colonized mice compared with GF mice. These effects were restricted to the colonic LP and were not evident in the MLN or spleen. The luminal concentrations of SCFAs, including acetate, propionate and butyrate, were higher in the cecum of high fiber-fed vs. low fiber-fed mice. *In vitro* and SPF studies showed that butyrate, and to a lesser extent propionate but not acetate, induced the differentiation but not proliferation of inducible Tregs. Feeding butyrylated high-amylase starch to *B. thetaiotaomicron*-monoassociated mice increased inducible IL-10-producing Tregs, but had no effect in GF mice, demonstrating that luminal bacteria are required to differentiate inducible colonic Tregs, even though *B. thetaiotaomicron* does not produce SCFA or induce Tregs on its own. This induction of Tregs was not mediated through MyD88. *In vitro* studies showed that butyrate acetylated histone H3 in Tregs. The investigators further demonstrated that dietary butyrate decreased colitis in a T cell-transfer model. Parallel studies by Smith et al. used conceptually similar, but somewhat different strategies to demonstrate that various SCFAs increased numbers of functional colonic Tregs (54). They first demonstrated decreased concentrations of colonic LP Tregs in GF mice associated with reduced concentrations of acetate, butyrate and propionate compared with SPF or gnotobiotic Altered Schaedler Flora-colonized mice. *In vivo* and *ex vivo* treatment of colonic Tregs from GF mice with propionate increased FoxP3 and IL-10 expression, but not TGFβ. Similar effects on colonic Tregs followed *in vivo* propionate treatment of SPF mice, but were not seen in the small intestine, MLN or spleen. Studies in SPF mice implicated signaling through the G-protein-coupled receptor (GPR)43, which most avidly binds propionate than other SCFAs. Propionate increased expression of Ffar2, the gene that encodes GPR43. Tregs were not induced in Ffar^−/−^ mice or in cells from these KO mice by *in vivo* or *ex vivo* exposure to propionate. Interestingly, acetate and propionate also stimulate colonic ILC3 proliferation, accumulation and IL-22 production in a Ffar2-dependent manner (60). Likewise, Tyagi et al. (61) stimulated numbers of FoxP3^+^ Tregs in small intestine Peyer's patches with butyrate. Other SCFA also have immunoregulatory activities. Pentanoic acid (pentenoate) is present in high concentrations in SPF, but not GF feces (62). Pentenoate induces IL-10 in lymphocytes by elevating glucose oxidation, a function mediated by increasing acetyl CoA for histone acetyl transferase and activating mTOR. Pentenoate activated regulatory B cells and intestinal TH_17_ cells during SFB colonization. Further evidence of protective effects of SCFAs is provided by preventing experimental colitis by administering butyrate-producing Clostridium subsets (43) and Lachnospiraceae (63). Furthermore, the systemic effects of luminal SCFAs are documented by direct administration of butyrate and propionate in drinking water preventing obesity and insulin resistance (64) and butyrate stimulating bone formation in a Treg-dependent manner (61).

##### Tryptophan metabolites (indoles)

Dietary L-tryptophan is metabolized to a number of immunologically active molecules that contribute to mucosal homeostasis (56, 65–69). Host enzymatic activities produce kynurenines and serotonin, while bacterial metabolism yields indoles, skatole, and tryptamine. These bacterial metabolites function as AhR ligands that activate a number of protective immune and epithelial pathways (56, 65, 67). Indole propionic acid (IPA) can additionally stimulate the pregnane X receptor (PXR) to improve the mucosal barrier (70). We describe several ways that gnotobiotic mice have been used to determine mechanisms of protection by these bacterial metabolites. Dodd et al. documented that *Clostridium sporogenes* can metabolize the three aromatic amino acids, tryptophan, phenylalanine and tyrosine to at least 12 compounds (55). They monocolonized GF mice with *C. sporogenes*, a species that can produce IPA, and an isogenic mutant with deletion of the fldC subunit of phenyllactate dehydratase that mediates IPA production. After 4 weeks, the ΔfldC mutant-colonized mice had no detectable cecal or serum levels of IPA and displayed increased mucosal permeability associated with increased peripheral blood concentrations of neutrophils, monocytes, CD4^+^ and CD8^+^ lymphocytes, as well as increased serum levels of *C. sporogenes*-specific IgG compared with mice colonized with the WT *C. sporogenes* strain. These biologic activities were PXR-dependent. Alexeev et al. reported that IPA was decreased in the serum of UC patients, with levels negatively associated with disease activity, and also in the serum and colons of SPF mice following DSS-induced colonic injury (71). Likewise, IPA is decreased in feces of patients with CD (51). Alexeev et al. showed that *E. coli* metabolites of tryptophan induced expression of the IL-10 receptor subunit (IL10R_1_) in colon cancer-derived T84 epithelial cells (71). GF mice monoassociated with *E. coli* WT K12 had elevated cecal levels of indoles and increased colonic epithelial IL10R_1_ expression, while *E. coli* K12 deleted for the tryptophanase A gene had negligible cecal indole concentrations and no induction of colonic IL10R_1_ gene expression over GF levels. Feeding IPA to SPF mice attenuated DSS-induced colitis. In addition, Cervantes-Barragan et al. showed that CD4^+^ CD8^+^ αα^+^ double positive (DP) T lymphocytes were absent in the small intestine of GF mice (25). These DP cells have regulatory functions that complement those of Tregs and promote tolerance to dietary antigens. The numbers of DP intraepithelial T cells were restored after colonizing GF mice with SPF microbiota, ileal microbiota from mice originating from Jackson Laboratories (JAX) with colonic *Lactobacillus reuteri* or with *L. reuteri* alone. *In vitro* experiments showed that *L. reuteri* cultured with tryptophan activated an AhR reporter cell line, while an AhR antagonist inhibited induction of *L. reuteri* supernatant-induction of DP T cells. Furthermore, *L. reuteri* produced indole-3-lactate acid. These studies show that *L. reuteri* produce an AhR ligand that differentiates DP T cells. In addition, Krishnan et el compared feces of GF vs. conventional mice to show that resident bacteria produce indole-3 acetate and tryptamine that provide protective signals for hepatic macrophages (72).

As mentioned earlier, impaired metabolism of tryptophan occurs in CARD9^−/−^ mice, a phenotype that could be transferred by FMT to GF mice (37). CARD9^−/−^ SPF mice, GF mice and CARD9^−/−^ FMT → GF mice had deficient levels of *E. coli* indole-3-acetic acid and decreased fecal AhR-stimulating activity compared with feces from SPF WT mice and WT FMT → GF mice. The enhanced susceptibility to DSS-induced colitis was reversed by administering an AhR agonist to CARD9^−/−^FMT → GF mice. Defective colonic expression of *Il22, RegIIIg*, and *RegIIIb* by GF, CARD9^−/−^, and CARD9^−/−^ → GF compared with WT mice and WT → GF mice was normalized by administering the AhR ligand (37). Supplementing the fecally-transplanted mice with three Lactobacillus strains (*L. murinus* CNCMI-5020, *L. reuteri* CNCMI-5022, and *C. taiwanensis* CNCMI-5019) that produce AhR ligands diminished colitis. IL22 is produced by intestinal LP immune cells, including ILC3 in response to AhR ligands and resident gut microbiota (28, 56).

Together, these studies indicate that multiple resident intestinal bacterial species produce indole metabolites of dietary tryptophan that stimulate mucosal homeostasis through protective effects on intestinal lymphocyte subpopulations, macrophages and epithelial barrier function.

### Strategies to Dissect Inflammatory Host Microbial-Immune Interactions

The same strategies used to understand mechanisms by which resident bacterial communities and components activate protective immune responses can also be used to identify the key microbial stimulants of effector immune responses and their functional pathways that mediate intestinal inflammation.

#### Using Genetic Models of IBD to Study Host-Microbe Interactions

The ability to genetically modify rodent models of intestinal inflammation and maintain them in gnotobiotic conditions provides an opportunity to investigate whether the inflammation exhibited by these models is dependent upon the microbiota. Early GF studies using HLA-B27/beta2-microglobulin transgenic rats found that lesions, such as gastric, duodenal and colonic inflammation and peripheral arthritis described in SPF HLA-B27/beta2-microglobulin rats were absent when studied in GF conditions (73). Similarly, mice with a disrupted interleukin-2 (IL-2) gene develop early onset, generalized clinical disease resulting in ~50% mortality. Furthermore, 100% of surviving mice exhibit striking colitis (74). However, GF IL-2-deficient mice showed no clinical abnormalities and exhibited only mild and significantly delayed onset of microscopic colonic inflammation. A very commonly used genetic model of IBD, the IL-10^−/−^ mouse was quite integral to early and continued work studying the relationship between the resident microbiota and colitis in the context of a genetically-predisposed host. Mice deficient in IL-10 developed spontaneous enterocolitis in a conventional housing setting, but inflammatory lesions were confined to the colon when housed in SPF conditions (12). This recognition of variable phenotypes induced by environmental factors led to use of GF and gnotobiotic IL-10^−/−^ mice to study the role of the resident enteric bacteria and their role in mucosal immune activation. Sellon et al. used GF IL-10^−/−^ mice on a 129S6/SvEv background and determined the microbiota was necessary for the induction of colitis in this line (12). GF IL-10^−/−^ mice lacked histologic evidence of colitis or other indicators of immune activation. Specifically, the total numbers of MLN lymphocytes were the same in both GF IL-10^−/−^ mice and IL-10^+/−^ mice with the proportions of CD4 and CD8 T cells of GF IL-10^−/−^ being similar to SPF WT mice. SPF IL-10^−/−^ mice had increased IgA, IgG1, IgG2a, and IL-12p40 measured in supernatants of colon fragment cultures compared with SPF WT mice. However, these differences were lost in the GF environment. Colonization of adult GF IL-10^−/−^ mice with fecal microbiota from SPF WT mice induced mild colitis and immune activation by 1 week that progressed to severe inflammation after 5 weeks. More recently, resident microbiota have been shown to impact Paneth cell and inflammatory response phenotypes in two mouse models of CD-susceptibility genes: *Irgm1*^−/−^ and *Atg16L1*^*HM*^ (75, 76). Although the vast majority of T cell-dependent, immune-mediated colitis models display a requirement for resident microbiota for expression of colitis (3), some exceptions exist. For example, Cominelli et al. reported that GF SAMP-1/YitFc mice retained ileitis, although the intensity of inflammation was diminished relative to SPF mice (77). Combined, these results highlight the important convergence of host genetic predisposition and resident microbiota, which significantly influences the exaggerated immune responses seen in IBD. Gnotobiotic rodent models are integral to deciphering the specific immune-microbial pathways involved in the pathogenesis of chronic immune-mediated intestinal inflammation by allowing investigators to manipulate and control all aspects of the environment. This manipulation can include monoassociation of GF mice with a single microbial culture or with a consortium of any number of cultured strains or communities collected from patients or other mice.

#### Fecal Microbial Transplant of IBD Patient Samples to Mice

Colonizing GF mice with human microbiota is a very powerful way to compare the function of microbiota collected from patients with inflammation to specimens collected from healthy individuals. Rather than simply correlating the overall microbial diversity and relative abundance of various microbial components with either healthy or inflammatory diseases, this approach allows the investigators to dissect out the particular pathways involved in activating inflammatory immune response and to design more mechanistic studies to identify particular microbial species or combinations for selective colonization or functional studies.

Nagao-Kitamoto et al. colonized GF mice with fecal microbiota collected from healthy human controls and patients with either CD or UC to determine whether IBD-associated dysbiosis is a cause or consequence of the intestinal inflammation. First, WT mice were inoculated orally with the donor stool (78). 16S rRNA sequencing of the fecal DNA collected from the murine recipients 2 weeks post inoculation showed at least some degree of dysbiosis in those mice that received IBD patient samples. The authors attributed the lack of statistically significant clustering of the UC and CD groups to the high intra-group variability. They next used the PICRUS algorithm to predict the functions of these sequenced bacteria and found that fecal microbiota collected from recipient mice retained the functional characteristics of the donor samples. More specifically, the feces of CD patients revealed an increased number of genes related to flagellar assembly and bacterial motility, and a fewer genes related to carbohydrate and bile acid metabolism than those of recipients of control feces. Functional analysis of UC patient feces showed an increase in glycolysis/gluconeogenesis-related genes and a reduction of genes associated with bacterial homeostasis. While there was some overlap between the CD and UC groups, overall, both metabolomic analysis of each group using CE-TOFMS and gene expression analysis found that each group exhibited distinct profiles, particularly as compared to the controls. For example, the level of luminal SCFAs and their expression level of propionate metabolism genes were both decreased in mice receiving human UC feces whereas succinate levels were increased compared to controls. Surprisingly, mice colonized with human CD feces showed increased luminal levels of SCFAs in comparison to the control group. Additionally the CD-associated group exhibited increased expression of epithelial host-defense responses, which the authors suggest could be due to the presence of pathobionts in the CD patient samples (44). Overall, however, the intestinal immune response of the CD-colonized group was significantly pro-inflammatory, with an inflammatory signature indicative of stimulation of mononuclear phagocytes and TH17 and TH1 signaling. Despite this inflammatory expression pattern, histopathologic lesions were absent from both control and study groups; however, this lack of intestinal pathology is consistent with other studies in WT mice (13, 14). This led the investigators to examine the impact of the IBD microbiota on colitis-prone IL-10^−/−^ mice. As expected, colonization of GF IL-10^−/−^ mice with human CD microbiota induced more significant histologic inflammation than those inoculated with control microbiota, consistent with the established hypothesis that manifestation of clinical IBD requires a genetic predisposition in addition to immune activation by intestinal microbiotia (3). Surprisingly, the UC microbiota did not induce colitis in the IL-10^−/−^ mice. The overall findings led the authors to suggest that IBD associated dysbiosis is not entirely secondary to the inflammation although it does play into the “vicious cycle of intestinal inflammation in IBD.” Gnotobiotic studies with native fecal communities and defined consortia of IBD-related strains clearly demonstrate both a primary drive and secondary effects of inflammation on bacterial community structure (14, 16, 78).

These studies demonstrate that human fecal samples can be readily placed into either WT or genetically-modified GF rodents to allow for controlled functional studies of immunologic, metabolomic and expression pathways impacted by the addition of such microbiota. Similarly, the study of host-microbe interactions can be further dissected down through the monocolonization of GF mice with species associated with IBD.

#### Monoassociation of GF Mice With Known IBD-Related Microbial Isolates

Our group has used monoassociated IL-10^−/−^ mice to determine that different resident bacterial strains convey different inflammatory phenotypes in the same host and to further show the importance of host genetic susceptibility and bacterial strain functional differences. Colonization of GF IL-10^−/−^ mice with either a murine fecal *E. coli* isolate, NC101, that was subsequently shown to have adherent-invasive characteristics, or the human oral isolate *Enterococcus faecalis* OG1RF led to colitis by histologic, clinic and immunologic measures (17). Furthermore, when murine colonic cultures were stimulated using these organisms, the colonic cultures secreted increased levels of IL-12p40 into the culture supernatant. Additionally, unfractionated MLN cells and APC-CD4^+^ cell cultures stimulated *ex vivo* with *E. coli* NC101 and *E. faecalis* lysates produced increased bacterial antigen-specific INFγ and IL-17 levels. Yet, importantly, the two bacterial strains induced very different colitis phenotypes, with *E. coli* stimulating relatively early onset cecal-predominant colitis evident after seven weeks of colonization. In contrast, the colitis induced by *E. faecalis* colonization was delayed in onset, was more severe, and primarily affected the distal colon. A key finding was that identically colonized WT mice had no evidence of inflammation or TH_1_ immune responses, directly demonstrating the key role of microbially-activated IL-10 in mucosal homeostasis. The importance of bacterial strain function is documented by differential results after colonizing GF IL-10^−/−^ mice with two adherent *E. coli* strains, murine NC101 and CUMT8 isolated from the ileum of a mouse with induced colitis and mild ileitis, compared with a standard lab strain K12 (79). The adherent-invasive *E. coli* (AIEC) strains each induced colitis and TH_1_ immune activation that was not seen with K12, despite near equal luminal concentrations of each strain. The AIEC strains attached to the colonic mucosa and invaded intact colonic epithelium, but the K12 strain did not, as measured by fluorescence *in situ* hybridization (FISH). Importantly, these strains are not pathogens, since WT monoassociated mice showed no evidence of colitis or TH_1_ immune activation.

Hoffmann et al. selected four human-derived bacterial and two yeast strains found to be either protective against or associated with dysbiosis in human IBD patients (80). As this section pertains to inflammatory models, we will focus here only on the inflammatory microbial strains utilized in the study: CD ileal-derived AIEC LF82, *Ruminococcus gnavus*, and the yeast *Candida albicans*. By monoassociating 6-week old female GF C3H/HeN mice with cultures of each organism, the authors were able to evaluate the impact of each organism through transcriptomic and histologic evaluation of the colon, host immune response, bile acid metabolism and short-chain fatty acid production. They confirmed that each organism successfully colonized the GF murine gut; yet, interestingly, they found that while the fungal organisms colonized the small and large intestines equally, the bacterial species more efficiently colonized the colon than the small intestine. The most notable findings in this study were the differences in host transcriptomics and cytokine production with the various monoassociations. These strains elicited a pro-inflammatory profile when studied in the monocolonized mouse. Additionally, colonic tissue from *E. coli* LF82 monoassociated mice displayed a striking level of upregulation of the indoleamine 2,3-dioxygenase gene (*Ido1*) in the colonic transcriptomic analysis. *R. gnavus* showed a lesser degree of upregulation of *Ido1* and other monoassociated colons lacked measurable differences. To validate the translatability of this finding, the authors inoculated a human HT-29 colonic epithelial cell line containing a luciferase reporter system with either *E. coli* LF82, *R. gnavus* or the non-CD associated *E. coli* MG1655. The *Ido* gene was upregulated only by *E. coli* LF82 and not the other bacteria. As IDO upregulation infers immunomodulatory effects mediated by the AhR pathway, the authors postulate that *E. coli* LF82 utilizes this mechanism as a protective strategy to evade the host immune response. Thus, further understanding of this pathway has potential therapeutic value. This study is an excellent example of how gnotobiotic mice are integral to examining of the ability of individual bacterial or fungal strains to induce inflammatory pathways associated with CD. Subsequently these pathways can be further validated in human cell lines. The ability of a single resident strain to activate aggressive immune responses is illustrated by elegant studies of Ivanov and Littman, who showed that monoassociation of GF C57BL/6 mice with SFB selectively activated ileal TH_17_ responses via activation of RORγT (81). The basis for these studies was the initial observation that mice purchased from different vendors: JAX and Taconic, displayed different frequencies of TH_17_ cells in their ilea. The authors identified that SFB accounted for these functional differences in SPF induction of TH_17_ cells.

#### Interactions of Resident Strains

Although much can be gained from the study of host-microbe interactions via bacterial monoassociation of gnotobiotic rodents to identify inflammatory mechanisms, it is also important to understand reciprocal interactions between relevant microbes and the impact of these interactions on both the microbiota and the host. GF rodents initially colonized with consortia of known microbial strains are then kept under gnotobiotic conditions to maintain the integrity of the microbial consortia. For example, Kim et al. selected two bacterial strains that are non-pathogenic in WT mice but cause inflammation in monoassociation of GF 129S6/SvEv IL-10^−/−^ mice, *E. faecalis* and *E. coli* (13), to ask whether the delivery of the two isolates would have an additive inflammatory impact. Dual-association led to a significantly more aggressive inflammatory signature as compared to their effects in monocolonization studies. Strikingly, the dually colonized IL-10^−/−^ mice had onset of histologically evident lesions and bacterial antigen-specific TH_1_ responses with an earlier age and much broader distribution as compared to the monoassociated IL-10^−/−^ mice, where *E. coli* caused proximal, cecal-dominated colitis and *E. faecalis*-induced distal disease (17). This study points out the value of colonizing gnotobiotic mice with more than one bacterial strain to better understand host-microbial interactions. More recently, larger defined consortia have been developed to better interrogate the function of individual microbial species in the context of a microbial community within gnotobiotic colitis models.

Eun et al. (14) selected 7 human-derived and IBD-related intestinal bacterial strains (SIHUMI) that are either altered in IBD patients or affect experimental colitis, are of human origin, have available genomic sequence, and are capable of forming a stable community in rodents. These included: the adherent/invasive *E. coli* LF82, *E. faecalis* OG1RF, *R. gnavus* ATCC 29149, *Bacterioides vulgatus* ATCC 8482, *Faecalibacterium prausnitzii* A2-165, *Lactobacillus plantarum* WCFS1, and *Bifidobacterium longum* subsp. *longum* ATCC 15707. The SIHUMI was used to colonize GF WT control and GF IL-10^−/−^ mice on both the 129S6/SvEv and C57BL/6 backgrounds. The two different background strains were utilized in this study to examine the impact of background strain on susceptibility to inflammation in mice deficient in IL-10. The investigators found that histologic colitis occurred after 12 weeks on both backgrounds; however, the degree of colitis was significantly higher on the 129S6/SvEv background as compared to the C57BL/6 background. Likewise, levels of IFNγ, IL-12p40, and IL-17 from the supernatants of unstimulated colonic tissue explants measured using ELISA at 6 and 12 weeks post-colonization were increased in IL-10^−/−^ mice on both the 129S6/SvEv and C57BL/6 backgrounds as compared to their WT counterparts. Furthermore, these levels were significantly higher in IL-10 deficient mice on the 129S6/SvEv background as compared to IL-10 deficient mice on the C57BL/6 background demonstrating that aggressiveness of colitis is significantly impacted by background strain. To address whether background strain influenced the bacterial composition over the length of the colonization, the relative abundance of each member of the SIHUMI consortia was measured via 16S rRNA gene based qPCR of feces, luminal contents and mucosal tissues collected at the end of 12 weeks of SIHUMI colonization in addition to feces collected at multiple timepoints after colonization. The analysis showed that the SIHUMI formed a stable community in the gnotobiotic conditions, but strain-related difference in composition was evident. The general trend was for *B. vulgatus* to predominate early on and for *E. coli* concentrations to decrease slightly over time. At the end of the study, the relative proportion of *R. gnavus* in 129S6/SvEv WT and IL-10^−/−^ mice exceeded that of *B. vulgatus* with the opposite occurring in the C57BL/6 WT and IL-10^−/−^. The presence of inflammation clearly impacted community composition, which was most evident in C57BL/6 IL-10^−/−^ and WT mice. Finally, MLN cells were stimulated via culture with lysate from each of the seven bacterial SIHUMI species or a combination lysate from the seven-strain inoculum. Unfractionated MLN cells from IL-10 deficient mice on both backgrounds produced the highest levels of both IFNγ and IL-12p40 when stimulated with *E. coli* lysate and yielded moderate levels of both markers when stimulated with the *R. gnavus* or combination lysate; however, the measured levels of IFNγ were higher in MLN cells from IL-10^−/−^ mice on the 129S6/SvEv background when compared to those on the C57BL/6 background. The highest levels of IL-17 resulted from stimulation by the *R. gnavus* or combination lysate and were again higher in the KO mice on the 129S6/SvEv background as opposed to the C57BL/6 KO mice. Thus, the authors concluded that *E. coli* and *R. gnavus* preferentially induced TH_1_ and TH_17_ effector immune responses in IL-10^−/−^ mice despite the relatively low concentrations of luminal *E. coli*. Thus, the ability to induce TH_1_ and TH_17_ cell responses upon stimulation with resident bacterial strains does not correlate with their relative abundance in feces or luminal contents, but may depend on antigenic properties of the organism or its availability to mucosal immune cells, with invasive strains having greater potential to induce host immune responses. This is an important consideration when drawing functional conclusions using 16S rDNA data. Also, the study pointed out that analysis of fecal DNA may not necessarily correlate with bacterial community structure of the mucosal tissue.

#### Studying Bacterial Genes Involved in Aggressive Function

Gnotobiotic mice also provide a very informative platform with which to study the bacterial genes that are differentially regulated in a state of inflammation. Jonathan Hansen's group has contributed to the understanding of bacterial genes involved in colonic inflammation using the AIEC murine *E. coli* isolate NC101. By monoassociating both GF WT and IL-10^−/−^ mice with NC101, they were able to generate a transcriptional profile of the bacterial genes that were differentially expressed in the cecal lumen of WT vs. colitic mice (82). The majority of upregulated genes were functionally associated with the bacterial stress response regulon, specifically *ibpB* and *ibpA* (*ibpAB*) genes encoding small heat-shock proteins thought to protect bacteria from reactive oxygen species (ROS) (83–85). To further define the role of these genes, they constructed a mutant NC101 strain lacking *ibpAB* (NC101Δ*ibpAB*), which was used to monoassociate GF WT and IL-10^−/−^ mice. They expected the lack of functional *ibpAB* to result in decreased growth of the mutant bacteria within the inflamed colonic lumens; however, they found the opposite to be true: luminal concentrations of NC101ΔibpAB was higher than WT NC101 in both the WT and IL-10^−/−^ mice and colonization with the mutated strain resulted in more severe colitis. Additionally, NC101Δ*ibpAB* exhibited increased survival within macrophages as well as elevated TNF secretion *in vitro*. A similar approach was later taken to evaluate the role of another set of stress-response genes found to be upregulated in their expression data: *gadA* and *gadB* (*gadAB*), to further define their role in bacterial survival within the inflamed colon (86). *GadAB* encode glutamate decarboxylase proteins within the glutamate-dependent acid resistance system 2 (87). This system protects the bacteria in acidic environments, such as during passage through the stomach (87). As with the NC101Δ*ibpAB*, it was expected that *in vivo* growth of NC101Δ*gadAB* would be reduced as compared to *WT NC101*. Again, the opposite was true as higher bacterial concentrations were present in the cecal and colonic luminal contents of both WT and IL-10^−/−^ mice colonized with NC101Δ*gadAB* than *WT NC101*. Colonization of WT and IL-10^−/−^ mice with NC101Δ*gadAB* also resulted in increased histologic inflammation, higher levels of IL-12/23p40 in colonic explants and increased levels of the proinflammatory cytokines IFNγ, IL-6, IL-17, and TNF-α (86). Recognizing that monoassociation may not adequately recapitulate the results of a bacteria in a complex community, they repeated these measures in WT and IL-10^−/−^ mice associated with NC101Δ*gadAB* in addition to a mixture of seven resident strains representative of the normal gut microbiota. Their findings were the same in the consortia as in the monoassociations and suggest that expression of *gadAB by NC101* in a colitogenic environment functions to attenuate the inflammatory response in monoculture or as part of a complex microbiota.

Likewise, Lengfelder et al. in Dirk Haller's group further demonstrated the importance of evaluating the pathogenic potential of a single microbial isolate not only via monoassociation of gnotobiotic mice but also as part of a complex bacterial consortia (88). They used *E. faecalis*, an organism shown to induce intestinal inflammation in monoassociated colitis-prone IL-10^−/−^ mice, to examine the functional role of this bacterium within the inflammatory response and as a member of a complex bacterial community. First they generated a RNA expression profile of *E. faecalis* in monoassociated gnotobiotic IL-10^−/−^ mice and found that, in an inflammatory colonic environment, *E. faecalis* responded with an expression profile primarily characterized by upregulation of genes associated with bacterial stress responses. Specifically, genes involved in ethanolamine (EA) utilization (*eut*) predominated among the top up-regulated genes. To further examine the role of ethanolamine utilization by *E. faecalis*, they monoassociated gnotobiotic IL-10^−/−^ mice with a mutant *E. faecalis* strain (Δ*eut*) that neither expresses the *eut* genes nor utilizes EA and found no difference in the colitogenic potential of the Δ*eut E. faecalis* strain as compared to that of the WT *E. faecalis*. To assess the gene expression profile of *E. faecalis* in the presence of other bacteria, they included either WT or Δ*eut E. faecalis* as a member of the SIHUMI microbial consortia we described earlier. Colonization of GF IL-10^−/−^ mice with Δ*eut E. faecalis* plus SIHUMI consortia induced significantly increased colitis as compared to colonization with the WT *E. faecalis* plus SIHUMI. This implied a protective effect of ethanolamine utilization by WT *E. faecalis* in the presence of a complex microbial community. Next, they isolated *E. faecalis* from the colons of SIHUMI-colonized IL-10^−/−^ mice to analyze the RNA expression profile as part of the consortia and compared the results with expression results from the *E. faecalis* monoassociation. Surprisingly, they found no overlap. In addition to providing new insights into how host responses and the resident microbiota influence *E. faecalis* function, this study highlights the importance of interrogating bacterial function *in vivo* not only using monoassociations, but also in the context of a complex bacterial community when designing association studies using gnotobiotic rodents.

#### The Impact of Diet on Intestinal Inflammation

The correlation between dietary intake and IBD is yet another opportunity to use gnotobiotic murine models to discern the underlying influence of diet and the intestinal microbiota on host inflammation and the mechanisms of these influences. Llewellyn et al. (89) examined the effect of 32 different diets with varied concentrations and sources of proteins, fat, digestible carbohydrates, and indigestible carbohydrates on intestinal inflammation in SPF and GF mice. Acute colitis was induced in these mice with DSS after 1 week on the specific diet with weight loss used as the primary measure of clinical disease. SPF mice receiving high protein diets, most specifically isolated soy protein and casein, experienced the most significant weight loss in response to DSS treatment. Consumption of diets high in palm oil, cellulose, and most profoundly, psyllium, led to significantly less DSS-associated weight loss in SPF mice. Conversely, methyl cellulose was the only fiber associated with increased weight loss in SPF mice. GF mice failed to display these diet-associated differences in weight loss, leading the investigators to further examine the role of the resident microbiota in their findings. The remainder of the study focused on the dietary components yielding the most significant weight loss: casein and psyllium. SPF mice on the high casein diet displayed increased intestinal permeability as measured by FITC-dextran challenge. Also, 16S rDNA sequencing of feces from non-DSS treated SPF mice receiving a high casein diet revealed significantly decreased bacterial diversity in the fecal microbiota of this group associated with a phylum-level increase in relative abundance of Bacteriodetes and decreases in Firmicutes. This casein-associated dysbiotic microbiota was inoculated into GF mice fed a control diet and then treated with DSS. However, the colitis phenotype was not evident in the latter experiment, implying that the high casein diet is also necessary to induce the DSS-induced increase in weight loss, perhaps by providing substrates for bacterial metabolism necessary to sustain the colitis-associated dysbiosis.

A related study also utilized gnotobiotic mice to examine the role of the source of dietary proteins on inflammatory pathways. Kostovcikova et al. (90) maintained SPF mice on either a standard protein level diet control (aCD) or a synthetic high-protein diets with the source of protein being either animal-derived casein (aHPD) or plant-based wheat gluten (pHPD). They found that mice fed animal protein had a significant increase in both numbers and activation levels of colonic Ly-6C^hi^ monocytes, yet the proportions of regulatory T cells or TH_17_ cells collected from mesenteric lymph nodes did not differ between groups. Cytokine analysis showed the animal protein-enriched group also had elevated expression of the inflammatory cytokines TNF-alpha and IL-1beta along with inducible NO synthase. Likewise, the animal protein group had a worsened progression of DSS-induced colitis. The investigators then fed the animal protein enriched diet along with a control diet to GF mice and found no differences in severity of DSS-colitis in the absence of the microbiota, confirming that the microbiota are necessary for the increased inflammation in mice fed the animal source protein diet. They then transferred gut microbiota from aCD or aHPD fed mice to GF mice being fed the standard diet (aCD) and once again subjected the mice to DSS-induced colitis. Similar to the previously described study, Kostovcikova et al. found that the microbiota from the aHPD was not sufficient to transfer increased severity of DSS-induced colitis, indicating that both the microbiota and the high level of animal protein are necessary for the increased inflammatory response.

This approach highlights the use of GF mice to separate the environmental influences of the microbiota from the feed content to determine the role of individual components on the inflammatory process. Of course, while the strength of using rodent models is our ability to control their environments, we must always consider the limitations of the use of a primarily herbivorous species of hind-gut fermenters to model the impact of an animal-based high protein or high fiber diet in humans. The innovative studies by Devkota et al. examined the role of dietary fat induced taurocholic acid and are detailed in a subsequent section (91).

Finally, GF mice provide a controlled environment to utilize multiple approaches described previously in this review to study mechanisms of IBD pathogenesis. In the next section, we provide examples of how the microbiota and dietary intake interact to impact defective epithelial barrier function.

#### Combining Gnotobiotic Approaches to Study Defective Epithelial Barrier Function

The luminal mucus layer forms a structural barrier between the intestinal epithelial cells and the luminal contents to serve as the first line of defense against luminal antigens and pathogens. In the small intestine, the mucus is comprised of a single layer, whereas, in the large intestine, there are two layers: an outer less-viscous layer that is host to limited bacterial species and a more-viscous inner layer, which is devoid of bacteria in the healthy state. Disruption of barrier function is associated with active IBD. More specifically, Johansson et al. found that bacteria penetrate the colonic mucosal layer in both UC patients and murine models of colitis (92).

Comparisons of GF and SPF mice determined that colonization with resident microbiota is essential to establish and maintain homeostatic intestinal epithelial barrier function. Furthermore, the establishment of homeostasis can be achieved through the conventionalization of formerly GF mice following a transient inflammatory response that occurs following the initial introduction of intestinal microbiota (93–97).

Increased numbers of sulfate-reducing bacteria (SRB), primarily those belonging to the genus *Desulfovibrio*, have been recognized in colonic biopsy samples and fecal samples from patients with active UC in contrast to samples taken during the quiescent phase or from healthy individuals (30, 98–100). Furthermore, elevated hydrogen sulfide (H_2_S) production has been associated with development of colitis. Thus, a functional connection between the bacteria and the elevated H_2_S levels leading to disruption of the mucus barrier is proposed (101). One mechanism by which H_2_S impacts barrier function is by blocking metabolic functions of butyrate in epithelial mitochondria. As mentioned above, butyrate and other SCFAs are the primary energy substrates for distal colonic epithelial cells (102, 103). Blocking this mitochondrial metabolic activity with hydrogen sulfide should decrease epithelial viability and diminish repair mechanisms after injury (104, 105). A mouse model of heme-induced epithelial damage and hyperproliferation showed a reduction in damage and epithelial proliferation in animals receiving broad-spectrum antibiotics, specifically by the loss of mucus barrier penetration via the elimination of sulfide-producing and mucin-degrading bacteria (101). Rey et al. colonized GF mice with a defined consortium of eight human gut microbial strains (31, 106) with and without the addition of *Desulfovibrio piger* GOR1, a SRB species with the highest prevalence among US adults included in their screen (107). Mice in both groups were fed either a diet low in fat and high in plant polysaccharides (LF/HPP) or a diet high in both fat and sugars (HF/HS). Sequencing of fecal microbial DNA 7 and 14 days post inoculation revealed that the relative abundance of *D. piger* was higher in mice consuming the HF/HS diet, suggesting that *D. piger* benefits from such diets- most likely due to an increase in free sulfate generation by the other members.

In addition to Desulfovibrio, another sulfite-reducing pathobiont, *Bilophila wadsworthia*, has also been identified as to potentiate colonic inflammation. Devkota et al. examined the effect of various dietary fats found in a typical western diet by comparing one high in saturated (milk-derived) fat (MF), another high in polyunsaturated (safflower oil) fat (PUFA), and a low fat (LF) diet (91). Diets were fed to SPF C57BL/6 mice and their fecal and cecal microbiota analyzed via 16S rRNA DNA sequencing. Overall, they found the PUFA and MF shifted the microbiota to a higher abundance of Bacteroidetes with a decrease in Firmicutes, whereas the LF diet promoted Firmicutes. A very interesting finding was the increase in relative abundance of the sulfite-reducing bacteria, *B. wadsworthia*, seen only in the MF group. Its appearance was of great interest given its rarity in healthy individuals but known immunogenic effect and association with intestinal inflammatory disorders. As this increased presence of *B. wadsworthia* did not seem to affect the WT mice, the investigators asked whether it would be immunogenic in rodent models of colitis by examining the impact of this species on the C57BL/6 mice treated with DSS to induce colitis and through its addition to IL-10^−/−^ mice. They found that the bloom of *B. wadsworthia* was apparent in the DSS-treated mice and the MF diet exacerbated the colitis. Likewise, *B. wadsworthia* increased the onset and incidence of colitis in the SPF IL-10^−/−^ mice and the increased severity of colitis with an inflammatory signature indicative of a TH_1_ immune response. A very important finding of this study was the exacerbation of colitis in the presence of the MF diet, and more strikingly, their inability to monocolonize GF IL-10^−/−^ mice in the absence of the high MF diet, further validating the important relationship between microbial colonization and dietary intake. Given the bloom of *B. wadsworthia* in the presence of a high MF diet and to the known proclivity of this bacteria to flourish in the presence of taurine-conjugated (TC) bile acids, the authors chose to further investigate a potential relationship between *B. wadsworthia* and bile acids. They stimulated *B. wadsworthia* in culture supplemented with fluid aspirates from the gall bladders of WT mice fed the LF, PUFA, and MF diets and found that only bile from the MF-fed mice resulted in a *B. wadsworthia* bloom. Next, they gavaged SPF IL-10^−/−^ mice TC, glycocholic acid, or control PBS and found significantly increased severity of colitis in the TC group only. Furthermore, they were able to establish monocolonization of *B. wadsworthia* and induce colitis in GF IL-10^−/−^ mice in the presence of TC. Together, these data support the role of dietary alteration of bile acids and their relationship with sulfate-reducing bacteria in the context of intestinal inflammatory response.

## Conclusions and Future Directions

Optimal use of gnotobiotic models can very efficiently dissect the functions of various components of the highly complex resident microbiota and their metabolic products that mediate mucosal homeostasis vs. intestinal inflammation. Gnotobiotic technology is particularly important to identify the dominant subsets of functional microbial species, the microbial genes and products that mediate these functions and the host pathways that transduce these microbial signals. Moreover, transfer of phenotype following FMT from human or murine donors can determine primary vs. secondary effects of microbial compositional changes, including the dysbioses that are associated with various inflammatory conditions. Answering this important “cause or consequence” controversy is convincingly addressed in many recent highly visible publications. Our targeted description of various strategies effectively used by different investigators in this review is designed to illustrate concepts; we apologize to the many investigators whose valuable work we are unable to cite due to space limitations.

Table 3 describes the balance of some of the dominant beneficial and potentially aggressive bacteria and their metabolites that mediate intestinal homeostasis or inflammation in normal or genetically-susceptible hosts, respectively. As with our examples used to illustrate strategies, this list is not comprehensive, but is derived from the gnotobiotic studies mentioned in this review.

**Table 3:** Balance of protective and potentially aggressive resident intestinal bacteria and metabolites.

**Protective**	**Potentially aggressive**
**Bacterial groups**	
Bacteroides species (*B. fragilis, B. vulgatus*)	Enterobacteriaceae (*E. coli, Klebsiella pneumonia*)
Clostridium species (select members of groups IV, XIVa, XVIII)	*Ruminococcus gnavus*
Lachnospiraceae	*Enterococcus faecalis*
*Faecalibacterium prausnitzii*	Fusobacterium species (*F. varium, F. nucleatum*)
Lactobacillus species (*L. reuteri*)	
**Bacterial metabolites or substrates**	
SCFA (butyrate, propionate, pentenoate)	↓SCFA
Indoles	Hydrogen sulfide
Sphingolipids	Sulfated bile acids
Secondary bile acids	Primary bile acids
	Ethanolamine
	Iron

Current gnotobiotic investigations are constrained by the incomplete sequence databases and paucity of validated resident fungal and viral isolates that limit expansion of these studies to members of the resident microbial communities beyond bacteria. Once additional fungal and viral isolates are available, strategies similar to those used for bacteria (Table 2) can be applied to explore their homeostatic or inflammatory functions. An additional unmet need is to improve the efficiency of engraftment following transplant of murine and human microbiota to GF recipients as addressed in the section describing FMT to transfer phenotype. A major shortcoming of rodent studies, including gnotobiotic investigations, is the general perception, particularly in the pharmaceutical industry, that observations in murine models do not readily translate human clinical conditions. We suggest several approaches to improve the clinical relevance of animal models (Table 4). A key strategy that is being incorporated by an increasing number of investigators is to “humanize” mice by transferring human complex microbiota to GF recipients. Numerous companies and investigators are developing consortia of live biotherapeutics agents that can be validated in carefully designed gnotobiotic studies that should provide evidence that murine gnotobiotic preclinical studies, when well-designed, reliably predict human outcomes. Studies in gnotobiotic mice identifying the characteristics of protective microbiota will hopefully be tapped to help design rational consortia of functional strains for therapeutic purposes. In parallel, further identification of functionally aggressive strains will provide targets for innovative approaches to restore normal microbial and metabolic balances in the intestine of patients with inflammation and dysbiosis that will convey long-term mucosal protection and homeostasis with limited toxicity.

**Table 4:** Strategies to improve translation of gnotobiotic murine model results to human application.

1. Use clinically relevant human microbial strains for selective colonization
2. Collect specimens from donors with important clinical disease phenotypes and matched healthy controls for FMT → “humanized” ex-GF mice
3. Validate results in at least two separate recipient GF strains or different inflammatory models, preferably with different mechanism of disease induction
4. For therapeutic studies, use treatment as well as preventive protocols
5. Cautiously interpret results

## Author Contributions

AR and RS contributed conceptually of the review and wrote sections of the manuscript. AO designed the figure. All authors contributed to manuscript revision, read and approved the submitted version.

### Conflict of Interest

The authors declare that the research was conducted in the absence of any commercial or financial relationships that could be construed as a potential conflict of interest.
